# Hybrid U-Net and Swin-transformer network for limited-angle cardiac computed tomography

**DOI:** 10.1088/1361-6560/ad3db9

**Published:** 2024-04-30

**Authors:** Yongshun Xu, Shuo Han, Dayang Wang, Ge Wang, Jonathan S Maltz, Hengyong Yu

**Affiliations:** 1 Department of Electrical and Computer Engineering, University of Massachusetts Lowell, Lowell, MA, 01854, United States of America; 2 Department of Biomedical Engineering, Rensselaer Polytechnic Institute, Troy, NY, 12180, United States of America; 3 GE Healthcare, 3000 N Grandview Boulevard, Waukesha, WI, 53188, United States of America

**Keywords:** limited-angle projections, cardiac computed tomography, image reconstruction, U-Net, Swin-transformer

## Abstract

*Objective.* Cardiac computed tomography (CT) is widely used for diagnosis of cardiovascular disease, the leading cause of morbidity and mortality in the world. Diagnostic performance depends strongly on the temporal resolution of the CT images. To image the beating heart, one can reduce the scanning time by acquiring limited-angle projections. However, this leads to increased image noise and limited-angle-related artifacts. The goal of this paper is to reconstruct high quality cardiac CT images from limited-angle projections. *Approach*. The ability to reconstruct high quality images from limited-angle projections is highly desirable and remains a major challenge. With the development of deep learning networks, such as U-Net and transformer networks, progresses have been reached on image reconstruction and processing. Here we propose a hybrid model based on the U-Net and Swin-transformer (U-Swin) networks. The U-Net has the potential to restore structural information due to missing projection data and related artifacts, then the Swin-transformer can gather a detailed global feature distribution. *Main results*. Using synthetic XCAT and clinical cardiac COCA datasets, we demonstrate that our proposed method outperforms the state-of-the-art deep learning-based methods. *Significance*. It has a great potential to freeze the beating heart with a higher temporal resolution.

## 1. Introduction

Computed tomography (CT) is a most utilized imaging modality in clinics and hospitals. In numerous CT applications, limited-angle scans occur due to various reasons such as accelerated imaging speed (Chen *et al* 2013). The limited-angle CT problem is to recover images when projection views collected over a limited angular range, which is typically less than 180° and represents a highly ill-posed problem. By successfully reconstructing images from a limited-angle dataset, we can image a dynamic object (e.g. a beating heart) within a shorter time window at a low radiation dose.

In a cardiac CT scan, even though the patient is instructed to hold breath to keep still, highly dynamic organs such as the beating heart are still in motion at a regular or irregular high frequency. Hence, in challenging cases cardiac CT images often exhibit motion artifacts due to insufficient temporal resolution (Reinhardt and Hoffman 1998). Since the temporal resolution is limited by the maximum speed of the rotating gantry, short-scan datasets are commonly used for image reconstruction, and they make it possible to select a cardiac phase for least motion artifacts. In many situations (e.g. atrial fibrillation), the current temporal resolution is not adequate to freeze the beating heart. This is because the optimal phase may be different for various vessels/segments of the coronary tree, and even if the best phase is selected, motion will still occur within the corresponding temporal window for a high heart beating rate. To further improve the temporal resolution of cardiac CT, advanced limited-angle image reconstruction is an excellent research topic of high clinical significance.

**Figure 1.**
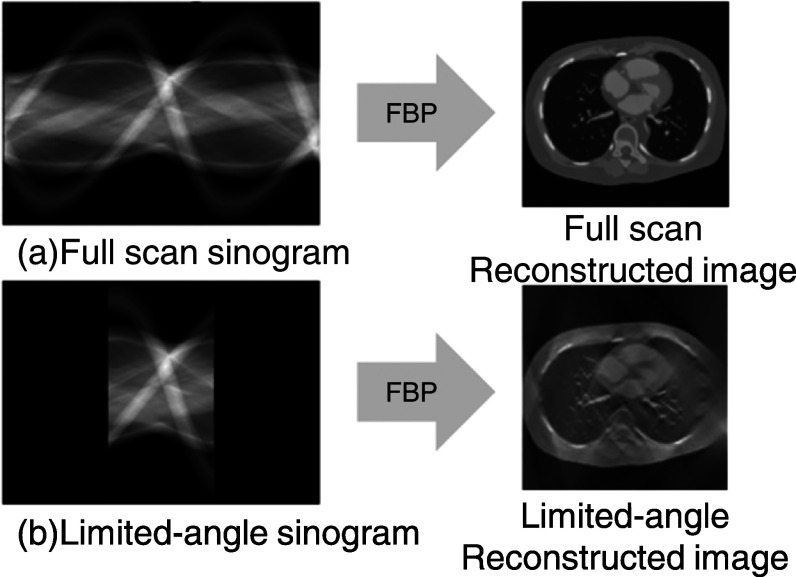
Illustration of streak artifacts in limited-angle reconstruction using FBP. The top row shows full-scan FBP reconstruction, while the bottom row is for limited-angle FBP reconstruction.

Directly applying a conventional image reconstruction algorithm (e.g. FBP) to limited-angle projections would result in poor images with severe streak artifacts relative to the full-scan reconstruction, as shown in figure 1. Previous studies attempted to address this issue using several approaches, such as explicit sinogram regression from limited view to full view. Under reasonable assumption when the data is insufficient, a data fidelity term and a regularization term were included into the objective function for minimization. For example, the CT image sparsity can be formulated into an imaging model to improve reconstruction results. The celebrated total variation (TV) regularization model is based on the piecewise constancy of the image, which means the sparsity of the discrete gradient transform. TV can suppress noise while preserving edges (Yu *et al* 2005, Wang *et al* 2023a). For example, TV can be coupled with SART (Andersen and Kak 1984) (simultaneous algebraic reconstruction technique) to enhance reconstruction performance. In the context of limited-angle reconstruction problems, these and other algorithms still encounter challenges in terms of missing details and persisting artifacts, yet at a high computational cost (Yang *et al* 2018).

In recent years, deep learning has been achieved great successes for computer vision tasks, such as image segmentation, image denoising, and super-resolution. Deep networks outperform traditional machine learning models and become the mainstream of medical imaging methodological development. The convolutional neural networks (CNNs) were first proposed to improve the presentation ability (Long *et al* 2015, Li *et al* 2022, Lei *et al* 2023, Morovati *et al* 2023, Li *et al* 2024). Then, generative adversarial network (GAN) (Anirudh *et al* 2018, Creswell *et al* 2018, Yang *et al* 2018, Li *et al* 2019, Yi *et al* 2019, Goodfellow *et al* 2020), U-Net (Ronneberger *et al* 2015, Chen *et al* 2017, Shan *et al* 2018, Chen *et al* 2021, Cao *et al* 2023, Li *et al* 2024), residual block (He *et al* 2016), *etc* were adapted for various medical imaging tasks. For example, U-Net (Ronneberger *et al* 2015) integrated skip-connections to facilitate detail retention. DD-Net (Zhang *et al* 2018) as a dense and deconvolution network increased the data quality by reusing features effectively. TomoGAN (Liu *et al* 2020) adopted the adjacent noisy images in a GAN framework. FBPConvNet (Jin *et al* 2017) added a residual connection from the first layer to the last layer to preserve shallow feature information. Beside the convolution-based model, the transformer model was successfully applied for computer vision tasks (Dosovitskiy *et al* 2020, Liu *et al* 2021, Dong *et al* 2022, Zamir *et al* 2022, Wang *et al* 2023b, 2023c, Chen *et al* 2024). The transformer depends on the attention mechanism instead of convolution operators to extract features and establish relationships among them. This model is much more powerful in gathering global contextual information than the convolution-based model.

In this paper, we propose a deep hybrid transformer model to improve the limited-angle cardiac CT image reconstruction. The transformer model tokenizes an image input into sequence patches and generates the global context. To increase the information context of features in the transformer block, the input is embedded into a U-shape network and coupled with the output of the transformer block, which is named as the UST block. This block integrates both highly detailed, localized information derived from convolution features and comprehensive global dependency contextual information extracted by the transformer. Our model is designed to synergize the convolution layer with a stacked sequence of UST blocks, with a long residual connection and followed by another convolution blocks to refine the results. By this design, our proposed approach allows for seamless integration of extensive global information from the transformer and local information from the CNN. Our proposed approach is systematically evaluated using a simulated XCAT (Segars *et al* 2010) dataset and clinical cardiac CT datasets, COCA dataset (2021) from Stanford center for artificial intelligence in medicine and imaging (COCA-coronary calcium and chest CT’s). The results show that our approach outperforms other state-of-the-art competing networks and hold a great promise to enhance the performance of limited-angle cardiac CT image reconstruction.

## 2. Related work

Our approach leverages the substantial achievements of transformer models and incorporates residual connections.

### 2.1. Vision transformer

Transformer (Vaswani *et al* 2017) is first proposed for transduction model using entirely on self-attention mechanisms to compute representations without using sequence aligned recurrent neural network (RNN) or convolution network. The transformer-based models (Vaswani *et al* 2017, Dosovitskiy *et al* 2020, Liu *et al* 2021, Wang *et al* 2021, Wu *et al* 2021, Dong *et al* 2022, Zamir *et al* 2022, Wang *et al* 2023b, 2023c) have achieved great success in many computer vision tasks, such as image classification, segmentation, inpainting, detection, *etc* vision transformer (ViT) (Dosovitskiy *et al* 2020) architecture divides a natural image into a sequence of non-overlapping patches with fixed length and learns inside knowledge by multiple consecutive multi-head self-attention modules to capture global features dependencies, and it can attain excellent results compared to the convolution network on image classification task. To further increase interaction between adjacent patches, a shift window (Swin) multi-head attention was developed by shifting the partition (Liu *et al* 2021). Some researchers develop conditional position encoding to improve the ViT data-efficient training (Touvron *et al* 2021), or use a pyramid structure to learn abstract representation in self-attention module (Dayan *et al* 2000, Wang *et al* 2021).

### 2.2. Residual block

Practices and theories that lead to shortcut connections have been studied for a long time. An early practice of training multi-layer perceptron (MLP) is to add a linear layer connected from the network input to the output. A few intermediate layers are directly connected to auxiliary classifiers for addressing vanishing/exploding gradients. ResNet (He *et al* 2016) first proposed the concept of residual block which is a fundamental component in deep neural network. It was introduced to address the degradation problem that arises when a neural network gets deeper. These connections bypass one or more layers, allowing the network to learn residual functions. The inclusion of residual blocks helps in training very deep networks by enabling smoother convergence during the training process. It mitigates issues like vanishing gradients and facilitates the learning of more abstract and complex features, leading to improved performance in various tasks such as image recognition, object detection, and natural language processing.

### 2.3. Hybrid CNN-transformer network

Introducing the convolution into vision transformers will improve the robustness while maintaining a computational and memory efficiency (Wu *et al* 2021). Many previous researchers proposed to combine CNN features and global features encoded by transformers, leading to hybrid CNN-transformer architectures. Swin-UNet (Cao *et al* 2023) is designed to apply a transformer for image segmentation by substituting all convolution layers with transformer blocks in the U-Net, replacing both down-sampling and up-sampling layers. TransUNet (Chen *et al* 2021) substitutes the convolution layers with consecutive transformer blocks on the deep feature maps. A study (Xiao *et al* 2021) attempts partitioning on the feature map rather than the image, suggesting that early convolutions can enhance the performance of transformers. SwinIR adds convolution layers and long shortcut connection to the transformer network (Liang *et al* 2021). Since U-Net achieves great success on image segmentation tasks, it can help the limited-angle images to restore major structural information. Those facts inspire us to develop a parallel network structure to combine U-Net and transformer blocks to learn structural features and details of limited-angle cardiac CT images. Long shortcut connection and convolution layer further enhance the image quality.

## 3. Methodology

In this paper, we propose a hybrid Swin-transformer-based image restoration model for limited-angle cardiac CT, which is named as U-Swin. The model is composed of three elements: Swin-transformer block, U-Net module, and skip connection. For the model architecture, we first use a convolution layer to extract the feature map, then we use a stack of residual U-Net Swin-transformer blocks for deep feature extraction, and a long skip connection is added to combine features and feed into the last convolution layer.

### 3.1. Limited-angle problem formulation

The CT data collection is a nonlinear process due to the polychromatic nature of the x-ray source (Kak and Slaney 2001). A common practice in CT adopts some linearization and discretization schemes that express the formation model as

\begin{eqnarray*}\begin{array}{c}g=Af+\varepsilon ,\end{array}\end{eqnarray*}

where $g\in {{\mathbb{R}}}^{\left(P\ast D\right)\times 1}$ is the measurement data, $P$ is angle number and *D* is detector cell number; $f\in {{\mathbb{R}}}^{\left(H\ast W\right)\times 1}$ is the image data, $H$ and $W$ are image height and width; $A\in {{\mathbb{R}}}^{\left(P\ast D\right)\times \left(H\ast W\right)}$ is the system matrix to represent the measurement procedure of the x-ray projection, and $\varepsilon \in {{\mathbb{R}}}^{\left(P\ast D\right)\times 1}$ is noise. Here, we use ‘$\ast $’ to represent one dimensional inner product and ‘$\times $’ to represent multiplication of different dimensions. We consider fan beam projection geometry, commonly employed in clinical CT reconstruction, where the scanning angle encompasses full $360^\circ $ rotation. For limited-angle reconstruction, we restrict the maximum scanning range as depicted in figure 1 for the full scan sinogram $g$ and limited-angle sinogram $g^{\prime} $ where only the limited-angle measurements are retained. We then address the inverse problem of reconstructing $f$ based on the given $g^{\prime} $ and $A.$

**Figure 2.**
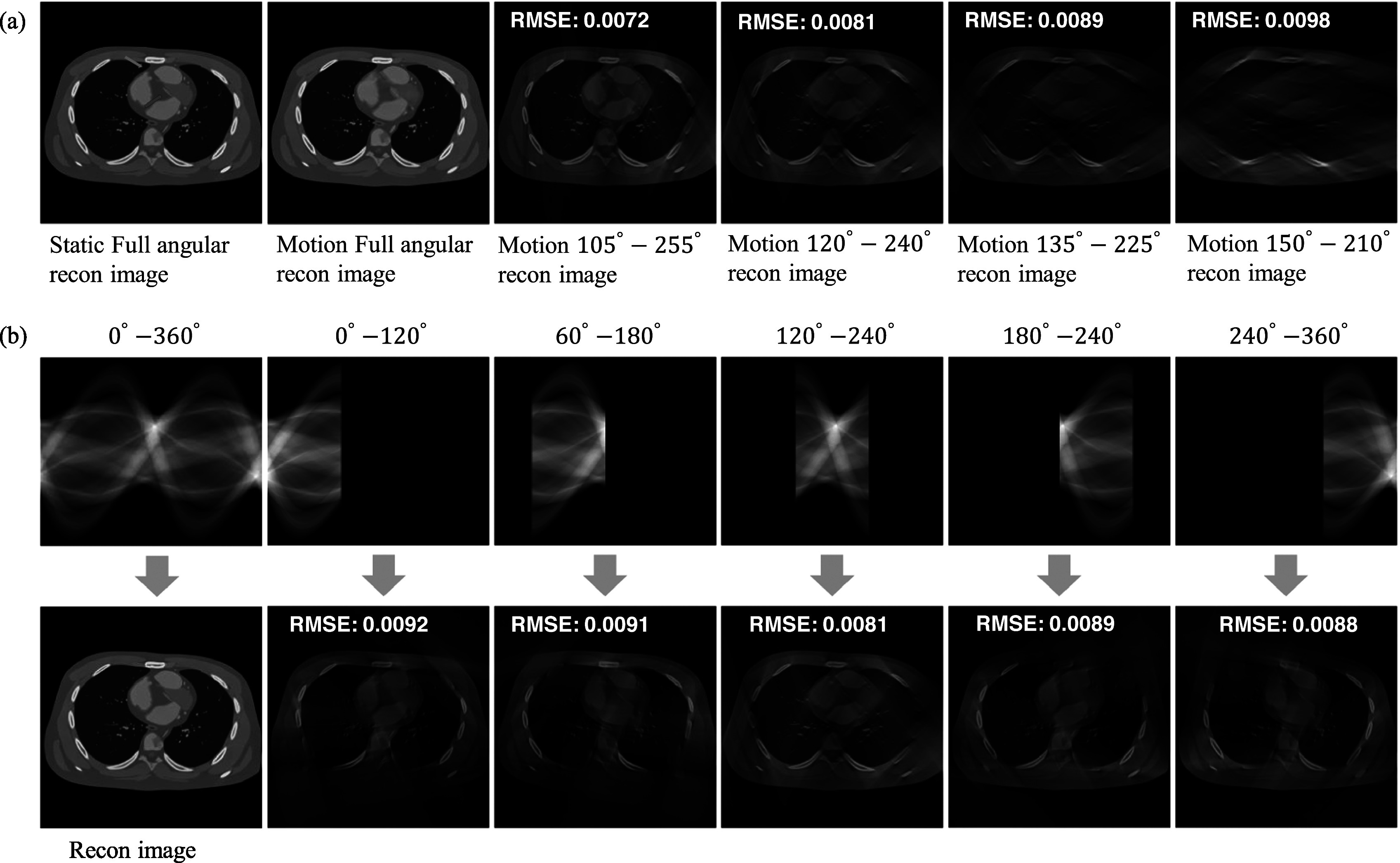
Limited-angle FBP reconstruction results with respect to different angular ranges. (a) Comparison of static image and motion images from different angular sizes. (b) Comparison of static reconstructed images using the same angular size but different starting angles.

In the simulation settings, a full scan assumes a gantry rotation time of 350 ms, while the nominal temporal resolution near the isocenter for a limited-angle 120° scan reconstructions is 117 ms. To optimize the experimental configuration, we present results concerning various angular sizes and starting angles. A static reconstructed image refers to images obtained by freezing the phantom during simulation, while a motion image denotes scenario where the phantom is dynamic during scanning. As illustrated in figure 2(a), a comparison between two reconstructed images—from full angular static projections and dynamic projections—reveals noticeable motion artifacts in areas of high activity, highlights blooming artifacts and blurring around vessels and the edges of organs, as indicated by the arrows. By reducing the scan angle range, it shortens scanning time and mitigates the motion artifacts. This effect is demonstrated through three FBP reconstructed images with varying angular ranges in figure 2(a). We can see that for the angular range of $150{^\circ },$ there are some blooming artifacts. Conversely, using a $90{^\circ }$ angular range data, it reduces motion artifacts but also loses many detailed information which subsequently increases the difficulty to restore the distorted image. In this task, we use $120{^\circ }$ and $90{^\circ }$ angular ranges to construct the limited-angle reconstructed image dataset and train the model. Regarding the starting angle, images have more artifacts in areas corresponding to the missing angles. As illustrated in figure 2(b), the quantitative results with starting angles of $0{^\circ },$ $60{^\circ },$ $120{^\circ },$ $180{^\circ },$ and $240{^\circ },$ with a consistent angular range of $120{^\circ },$ indicate that $120{^\circ }$–$240{^\circ }$ degree angular range has a smaller RMSE score for the human anatomy. Consequently, we select $120{^\circ }$–$240{^\circ }$ and $135{^\circ }$–$225{^\circ }$ angular ranges for our experiments, corresponding to $120{^\circ }$ and $90{^\circ }$ angular ranges, respectively.

### 3.2. Hybrid convolution transformer network

The pipeline of our proposed U-Swin is to convert the sinograms to CT images by FBP algorithm, then using deep learning technique to denoise and complete the CT images and improve their quality. Our network is constructed based on a residual U-Net and Swin-transformer. The U-Net is one of the CNN models proposed for image segmentation. Residual learning can make a network converge faster and more efficiently. It trains the network to learn the difference between the ground truth and the input data. This network consists of three modules: shallow feature extraction, deep feature extraction, and high-quality image reconstruction.

**Figure 3.**
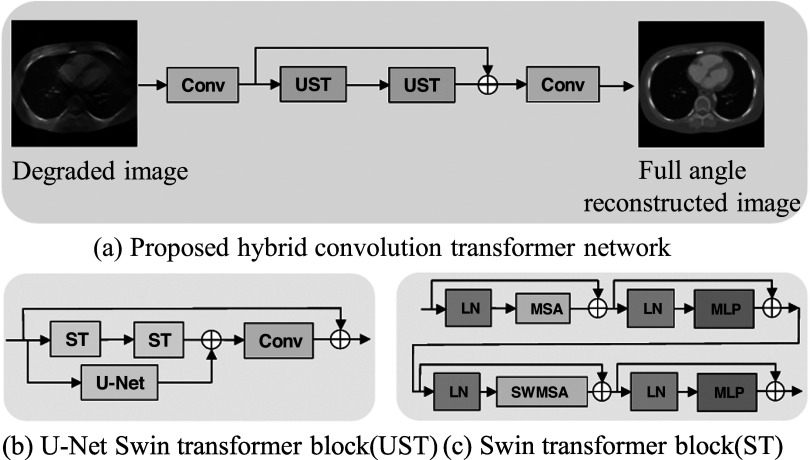
The framework of the proposed U-Swin network. In (a), UST is Swin-transformer U-Net block, the input is low quality FBP results from limited-angle projections, and the label is high quality FBP results from full view projections without motion. (b) is the structure of UST block, where ST represents a Swin-transformer block. (c) is the structure of ST block, where LN is layer normalization, MSA is multi-head self-attention, MLP is multilayer perceptron, and SWMSA is shift window multi-head self-attention.

As shown in figure 3(a), low-quality image reconstructed from limited-angle projections serves as an input ${I}_{\mathrm{LA}}\in {{\mathbb{R}}}^{H\times W},$ where *H* and *W* are image height and width. We use a convolution layer ${\mathrm{Conv}}^{C}$ to extract feature maps, the kernel size is $3\times 3,$ and $C$ represents the channel number. The convolution layer is good at early visual processing, leading to more stable optimization and better results. It also provides a simple way to map the input image space to a higher dimensional feature space. Then, we obtain a feature with higher dimension $F\in {{\mathbb{R}}}^{H\times W\times C}.$ This processing step will help to improve the final reconstruction results. UST represents Swin-transformer blocks, and two UST blocks ${\mathrm{UST}}_{1}$ and ${\mathrm{UST}}_{2}$ are consecutively connected after the convolution layer. Then, a residual connection is used to combine the early-stage features and the deep features. Next, we use a convolution layer with one channel ${\mathrm{Conv}}^{1}$ to improve the local features. Using a convolutional layer at the end of feature extraction can bring the inductive bias of the convolution operation into the Transformer-based network and lay a better foundation for later aggregation of shallow and deep features.

\begin{eqnarray*}\begin{array}{l}{I}_{\mathrm{out}}={\mathrm{Conv}}^{1}\left(\left({\mathrm{UST}}_{2}\left({\mathrm{UST}}_{1}{\mathrm{Conv}}^{C}\left({I}_{\mathrm{LA}}\right)\right)\right)+{\mathrm{Conv}}^{C}\left({I}_{\mathrm{LA}}\right)\right).\end{array}\end{eqnarray*}

In the experiment for image reconstruction, we train the network by minimizing the mean square loss.

\begin{eqnarray*}\begin{array}{l}\mathrm{Loss}=\displaystyle \frac{1}{N}{\parallel {I}_{\mathrm{out}}-{I}_{\mathrm{FV}}\parallel }_{F}^{2},\end{array}\end{eqnarray*}

where ${I}_{\mathrm{FV}}$ represents the ground-truth that is reconstructed by FBP/FDK from full view motionless projections, and $N$ is the total pixel number.

As shown in figure 3(b), the Swin-transformer U-Net block is a residual connection with parallel Swin-transformer layers and U-Net. Given an input feature map ${f}_{\mathrm{in}},$ we parallelly feed it into the Swin-transformer layer $\mathrm{ST}$ and U-Net layers. Then, we extract the latent features and feed into the convolution layer $\mathrm{Conv}.$ Finally, we add the residual connection to get the output feature ${f}_{\mathrm{out}}.$

\begin{eqnarray*}\begin{array}{l}{f}_{\mathrm{out}}=\mathrm{Conv}\left(U\left({f}_{\mathrm{in}}\right)+{\mathrm{ST}}_{2}\left({\mathrm{ST}}_{1}\left({f}_{\mathrm{in}}\right)\right)\right)+{f}_{\mathrm{in}}.\end{array}\end{eqnarray*}

In this block, U-Net is used to gather more structural information, and Swin-transformer layer can integrate better global spatial information. The Skip connection layer can aggregate distilled features to improve the shallow features.

**Figure 4.**
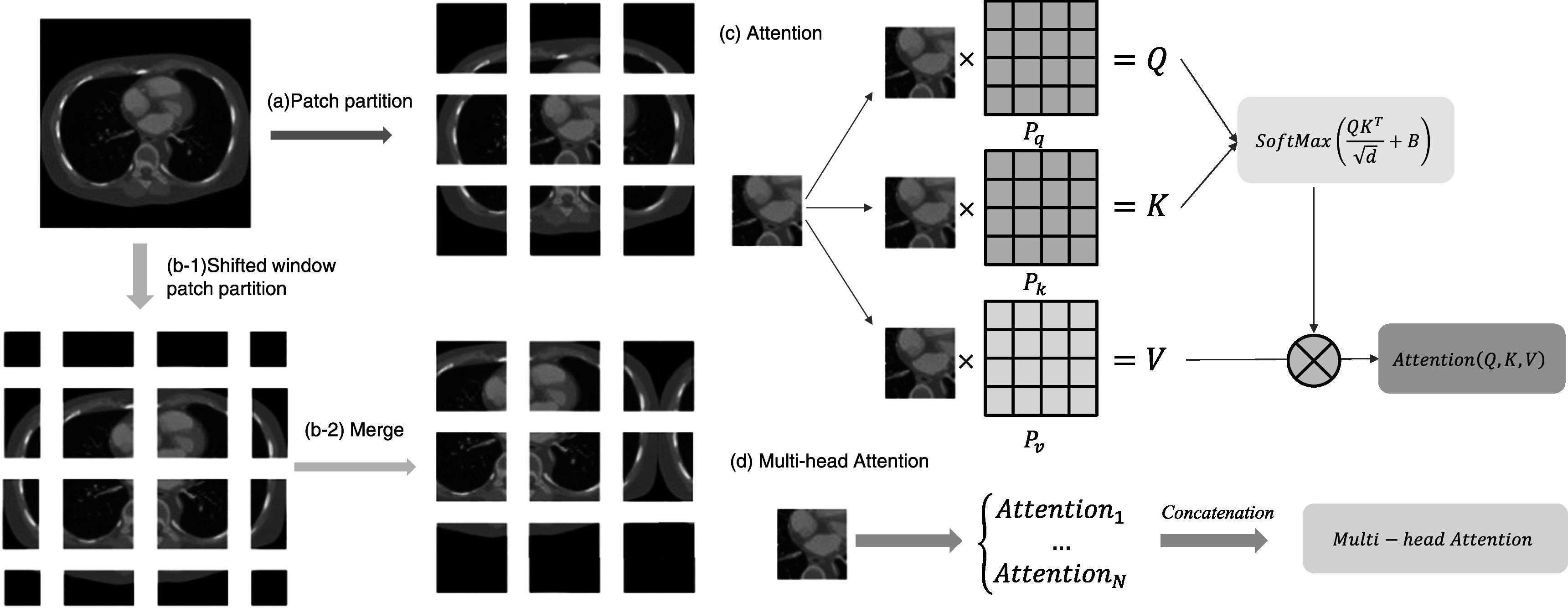
Illustration of the Swin-transformer procedure. (a) Illustration of patch partition, (b) shift window patch partition, (c) attention mechanism, and (d) multi-head attention.

In figure 4(c), we show a standard Swin-transformer layer that consist of two consecutive transformer blocks. The first is a window multi-head attention block, and the second is a window shift multi-head attention block. Each block includes layer normalization, skip connection and two-layer multilayer perceptron, and ReLU layer. As pointed out in the first work of Swin-transformer (Liu *et al* 2021), the shifted window partitioning approach introduces the connections between non-overlapping windows in previous layer, and it is effective for feature extraction. As shown in figure 4(a), given an input feature size of $H\times W,$ the transformer first reshapes it into $\displaystyle \frac{HW}{{M}^{2}}\times {M}^{2}$ features by partitioning the input into non-overlapping $M\times M$ local windows, where $\displaystyle \frac{HW}{{M}^{2}}$ is the total number of windows. However, when the partition is fixed for different layers, there is no connection across local windows. Therefore, as shown in figure 4(b), regular and shifted window partitioning are used alternately to enable cross-window connections, where shifted window partitioning means shifting the feature by M/2 pixels before partitioning. Then, it computes the self-attention on each window, as shown in figure 4(c). For a local window feature $X\in \,{{\mathbb{R}}}^{{M}^{2}},$ by using projection matrices ${P}_{q},\,{P}_{k},\,$ and ${P}_{v};$ $Q,K,$ and $V$ are computed as:

\begin{eqnarray*}\begin{array}{c}Q=X{P}_{q},K=X{P}_{k},V=X{P}_{v}\,.\end{array}\end{eqnarray*}

The self-attention process is as following:

\begin{eqnarray*}\begin{array}{l}\mathrm{Attention}\left(Q,K,V\right)=\mathrm{SoftMax}\left(\displaystyle \frac{Q{K}^{T}}{\sqrt{d}}+B\right)V,\end{array}\end{eqnarray*}

where $Q,K,V\in \,{{\mathbb{R}}}^{{M}^{2}\times d}$ are the query, key, and value matrices, $d$ is the dimension and ${M}^{2}$ is the number of features in a window, and $B$ is relative position bias $B\in \,{{\mathbb{R}}}^{{M}^{2}\times {M}^{2}}.$ Next, a MLP that has two fully connected layers with GELU non-linearity between them is used for further feature transformations. The LayerNorm (LN) layer is added before both the MSA and MLP, and the residual connection is employed for both of the modules. The whole process is formulated as

\begin{eqnarray*}\begin{array}{l}X=\mathrm{MSA}\left(\mathrm{LN}\left(X\right)\right)+X,X=\mathrm{MLP}\left(\mathrm{LN}\left(X\right)\right)+X.\end{array}\end{eqnarray*}

The network encoder module is mainly used to extract the feature information of the limited-angle sinogram, to down-sample the lower-level mask and images layer by layer, and to learn a high-level compact latent feature.

## 4. Experimental design and results

In this section, we aim to present the experimental validation and highlight the results of our study. Our presentation starts with an introduction to the datasets. We then offer a detailed description of the parameters and the experimental setup. Our evaluation involves a comparative analysis of our method against the state-of-the-art approaches, concentrating on addressing the limited-angle CT reconstruction. Finally, an ablation study will showcase the effectiveness of our model.

### 4.1. Datasets

#### 4.1.1. XCAT dataset

We use the 4D extended cardiac-torso (XCAT) phantom version 2 to generate realistic projection data to simulate a cardiac CT imaging procedure. This widely utilized multimodality phantom is developed at the Duke University and is described in detail in (Segars *et al* 2010). The XCAT can output a 4D phantom with attenuation coefficients to mimic a patient with a beating heart which is close to a realistic dynamic cardiac CT imaging situation. Phantoms are generated based on a set of pre-defined parameters, including spatial resolution, temporal resolution, respiration rate, and heart rate. After generating the digital phantoms, we use XCAT’s CT projector to generate the cone-beam CT projections. The CT projection simulation is controlled by some key parameters, such as the distance from the object to the source, the distance from the object to the detector, the detector array size, the x-ray source energy spectrum, and the beam half-fan angle. These key parameters are configured to mimic a representative GE CT scanner. After generating the projection data using a circular scan, the standard Feldkamp–Davis–Kress (FDK) algorithm is employed to reconstruct the 3D volumetric image for each phase and scaled into Hounsfield unit (HU). Based on the tuned parameters, simulated images are generated for 10 patients both on dynamic phantom and static phantom. We reconstruct images at phases from 20% to 80% with an interval of 3% (Xu *et al* 2022). Each simulated human phantom yields 21 different motion-blurred phases. For each phase, we apply the standard FDK algorithm to generate 32 image slices. For dynamic data, we select 120 degree-range and 90 degree-range views to reconstruct limited-angle images. Thus, we generate two cardiac phantom image datasets, each of them contain 6720 motion blurred limited-angle images and the corresponding 6720 static ground-truth images. The reconstructed images have 256 $\times $ 256 pixels. We use the 10-fold cross-validation method to show the rigorous of model. In each experiment, we select images from nine numerical patients as the training dataset, and the images from the last numerical patient as the testing dataset.

#### 4.1.2. Stanford COCA dataset

This dataset contains gated coronary calcium (COCA) CT images provided by the Stanford center for artificial intelligence in medicine and imaging (AIMI) (COCA- coronary calcium and chest CT’s). We select 4788 cardiac CT images with size of $512\times 512.$ It includes 84 patients, and 57 images are for each patient. 74 patients’ images are used as the training dataset, and the rest 10 patient’s images are for testing dataset. The limited-angle reconstructed images are from $120{^\circ }$ and $90{^\circ }$ projections.

### 4.2. Experimental setting

We implement the proposed U-Swin network with PyTorch and use one NVIDA 2080Ti GPU for training. We employ the following setting: batch size = 1, number of epochs = 100, Adam is used to optimize the model, and the learning rate is set at ${10}^{-5}.$

The experiments include a comparison of different methods using the same training and testing datasets, and the images are reconstructed from angular ranges of $120{^\circ }$ and $90{^\circ }.$ The comparison methods include SART-TV (Sidky *et al* 2008), FBPConvNet (Jin *et al* 2017), TomoGAN (Yang *et al* 2018), DDNet (Zhang *et al* 2018), U-Net (Ronneberger *et al* 2015), and SwinIR (Liang *et al* 2021). Two representative slices from the testing dataset are shown to enable visual comparison of the methods, including the major vessels and bone structures. Both qualitative and quantitative analysis are performed. We also perform ablation study of our proposed U-Swin network to validate the effectiveness of each module and compare the performance using different number of blocks. Finally, we evaluate the performance of different models on the COCA dataset through qualitative analysis.

### 4.3. Evaluation metric

Two metrics are employed for evaluation of image quality, root of mean square error (RMSE) and structural similarity (SSIM). RMSE measures the difference between the reconstructed image and ground-truth image. A smaller RMSE value generally suggests a high-quality reconstructed result.

\begin{eqnarray*}\begin{array}{l}\mathrm{RMSE}=\sqrt{\displaystyle \frac{1}{N}\displaystyle \sum _{N}^{i=1}{({Y}_{i}-{\hat{Y}}_{i})}^{2}},\end{array}\end{eqnarray*}

where *N* is the number of data points, ${Y}_{i}$ is the $i\mathrm{th}$ observed values of the variable being predicted, and ${\hat{Y}}_{i}$ is the $i\mathrm{th}$ being the predicted value.

The SSIM is a method for predicting the perceived quality of digital television and cinematic pictures, as well as other digital images and videos. SSIM is used for measuring the similarity between two images. It ranges between −1 and 1 with a value approaching 1 meaning that the two images are more identical.

\begin{eqnarray*}\begin{array}{c}{\mu }_{x}=\displaystyle \frac{1}{N}\displaystyle \sum _{i=1}^{N}{x}_{i},{\mu }_{y}=\displaystyle \frac{1}{N}\displaystyle \sum _{i=1}^{N}{y}_{i},\end{array}\end{eqnarray*}

\begin{eqnarray*}\begin{array}{c}{\sigma }_{x}=\,{\left(\displaystyle \frac{1}{N-1}\displaystyle \sum _{i=1}^{N}{({x}_{i}-{\mu }_{x})}^{2}\right)}^{\displaystyle \frac{1}{2}},{\sigma }_{y}=\,{\left(\displaystyle \frac{1}{N-1}\displaystyle \sum _{i=1}^{N}{({y}_{i}-{\mu }_{y})}^{2}\right)}^{\displaystyle \frac{1}{2}},\end{array}\end{eqnarray*}

\begin{eqnarray*}\begin{array}{c}S\left(x,y\right)=\displaystyle \frac{\left(2{\mu }_{x}{\mu }_{y}+{C}_{1}\right)\left(2{\sigma }_{x}{\sigma }_{y}+\,{C}_{2}\right)}{\left({\mu }_{x}^{2}+{\mu }_{y}^{2}+{C}_{1}\right)\left({\sigma }_{x}^{2}{\sigma }_{y}^{2}+{C}_{2}\right)},\end{array}\end{eqnarray*}

where *N* is the number of pixels in the image and ${x}_{i},{y}_{i}$ are the pixel values. ${\mu }_{x},{\mu }_{y}$ are the image mean values, ${\sigma }_{x},{\sigma }_{y}$ are the image standard deviations, ${\sigma }_{xy}$ is the cross-covariance for image $x,y,$ and $S\left(x,y\right)$ is to compare the structural similarity.

Noise power spectrum (NPS) (Wang *et al* 2020) is also used to compare the reconstructed CT images and the ground-truth images in the testing set. The NPS is calculated as follows:

\begin{eqnarray*}\begin{array}{l}\mathrm{NPS}\left(\mathrm{img},\mathrm{ref}\right)=\mathrm{log}\left({\left|\mathrm{FFT}\left(\mathrm{img}-\mathrm{ref}\right)\right|}^{2}\right),\end{array}\end{eqnarray*}

where $\mathrm{img}$ and $\mathrm{ref}$ are the limited-angle reconstructed CT image and full scan CT image, respectively, and FFT is the 2D Fourier transform.

### 4.4. Limited-angle reconstruction results

**Figure 5.**
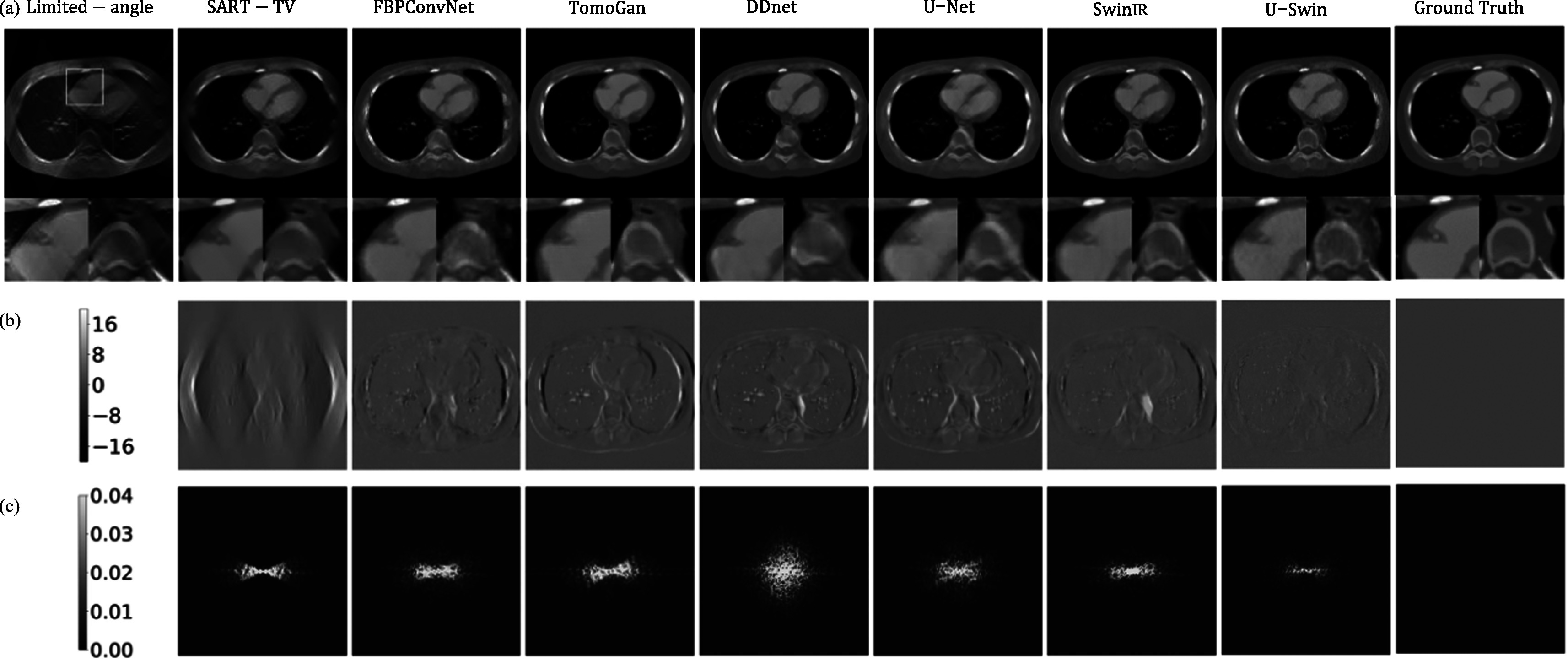
Reconstructed results of a representative image slice from patient 153 (phase 40, slice 21). (a) Visual comparison of cone-beam CT reconstructions generated from 120 degree viewing angles using different methods. The last column shows the ground truth (GT) images. Each reconstructed image is accompanied by two magnified ROIs (regions of interest). The display window is [−800, 1000] HU. (b) The difference maps between reconstructed and ground truth images of each method in terms of HU. (c) The 2D noise power spectrum (NPS) maps of different methods. The inner area of the maps represents low-frequency noise components, while the outer area represents high-frequency noise components.

**Figure 6.**
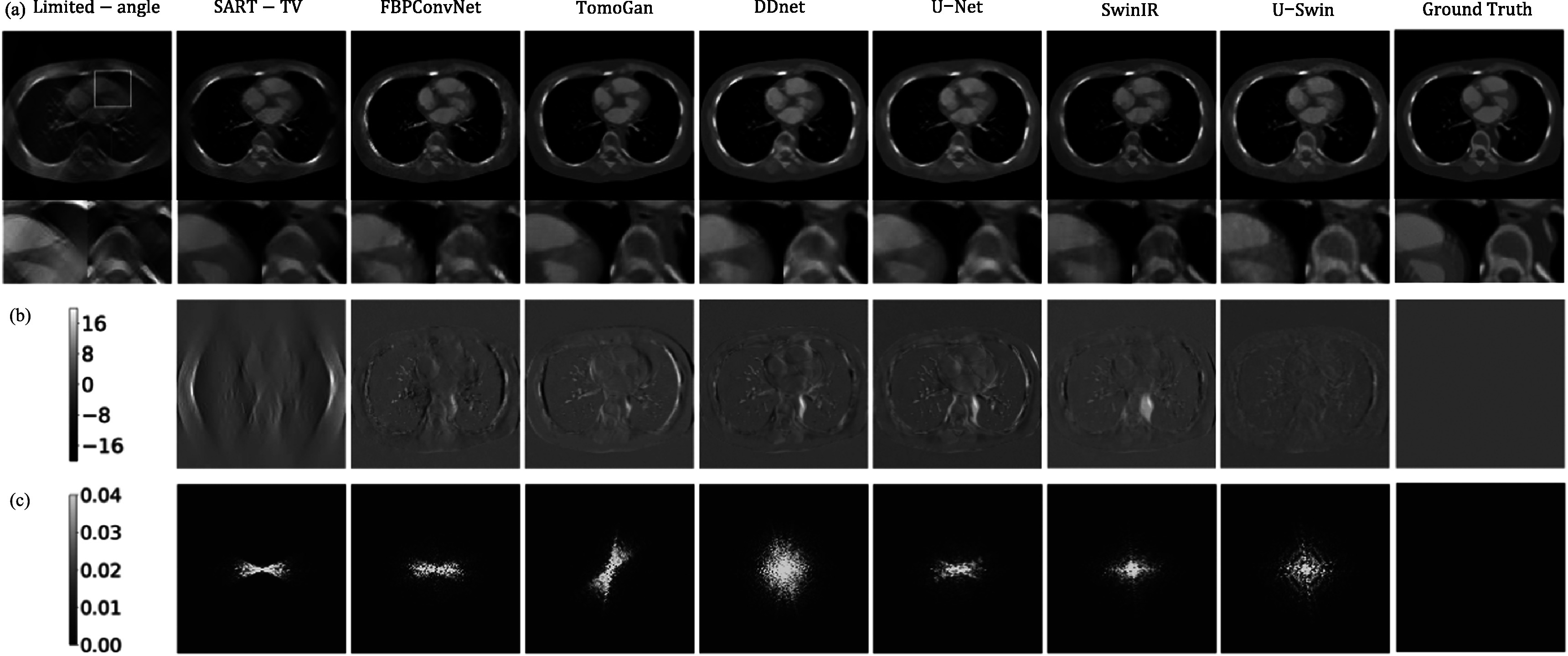
Same as figure 5 but for patient 153 (phase 40, slice 10).

In cardiac CT images, our primary focus lies on the reconstructed image quality of vessels, particularly the three major arteries—right coronary artery (RCA), left circumflex artery (LCX), and left anterior descending artery (LAD)—as they hold utmost significance for diagnosis. Two representative slices are presented. While figure 5 corresponds to RCA, figure 6 corresponds to LCX. In this part, we compare the performance of FDK, convolution-based model DDNet, U-Net, FBPConvNet, GAN-based model TomoGAN, and our proposed U-Swin model. The limited-angle reconstruction images, full view reconstruction images, and reconstructed images using different networks are shown in figures 5(a) and 6(a).

Due to the limited scanning range of projections, there are severe streak artifacts appearing in the FDK reconstruction results, only a coarse image is recovered, and it significantly compromises the edge and texture information. As shown in figure 5(a), the region of interests (ROIs) indicated by the yellow and red boxes are magnified under each image. Although all the deep learning-based methods can suppress part of streaking artifacts, there are notable differences. According to the results, SART-TV, a traditional iterative reconstruction method to leverage prior sparsity knowledge total variation (TV), could recover some missing information. However, it still falls short in capturing a significant amount of detailed information concerning the location of the missing angle, and it still has severe streak artifacts. For all other convolution-based or GAN models, while they can remove a significant portion of streak artifacts, they will either compromise the edge information or introduce some noise in the final results. The FBPConvNet lacks of ability to effectively suppress the noise on the boundary and the bony structure, and some boundaries have distortions. The U-Net restores a great amount of edge information compared with other convolution-based models. It maintains piecewise smoothness better than the TomoGan. Nonetheless, it still lacks capability to preserve certain detailed localized information, such as vessel information. The organs and bony structures contain more noise. For the SwinIR transformer model, although it can reliably reconstruct structures, it is hard to restore the lost information. As shown in the red ROI, the tissue regions are not successfully restored.

**Figure 7.**
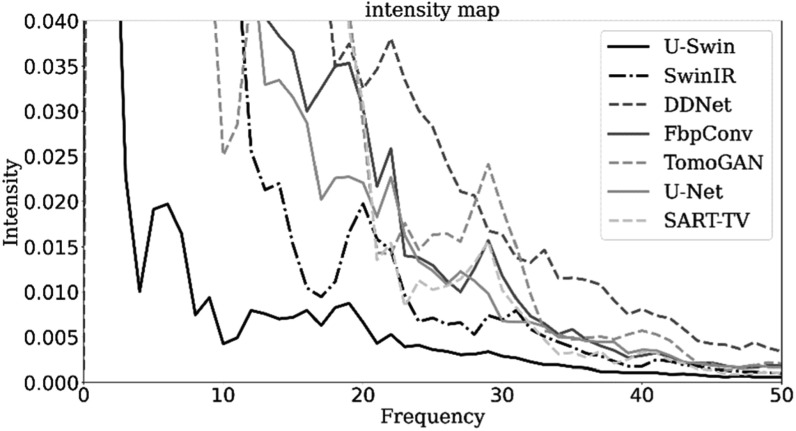
Noise power spectrum plots of different methods for image slice from patient 153 (phase 40, slice 21). The frequency level is represented on the *x*-axis, while the intensity level is indicated on the y-axis.

The difference maps in figure 5(b) present an alternative perspective for comparison. Both the convolutional-based model and the transformer-based model notably reduce the streak artifacts. Nevertheless, noticeable noise persists in the central region, along with some discrepancies at the borders. Specifically, in the left and right middle regions where the artifacts are most pronounced, the convolution-based model manages to recover some localized information. In contrast, the GAN model’s performance is limited as it can generate images resembling reality but lacks deterministic similarity to the ground-truth image. Notably, the hybrid convolution-transformer model performs impressively, exhibiting minimal noise and artifacts across the entire image. The noise power spectra maps in figure 5(c) show high frequencies at the boundary and low frequencies in the middle. We also compute the average NPS for different frequencies, corresponding to different concentric circles in the 2D map, and obtain a NPS plot for each image reconstruction method (see figure 7). One can see that our proposed method demonstrates lower values in both high and low-frequency domains, indicating superior image quality. In general, the proposed method significantly enhances the quality of reconstructions, obtaining a closer result to the ground-truth than other methods. The result demonstrates a reduction in artifacts at the edges and a more precise reconstruction of dynamic objects. Specifically, the method effectively restores vessel information, creates more compact bony structures, and accurately preserves sharp boundaries to ensure well-defined edges.

**Figure 8.**
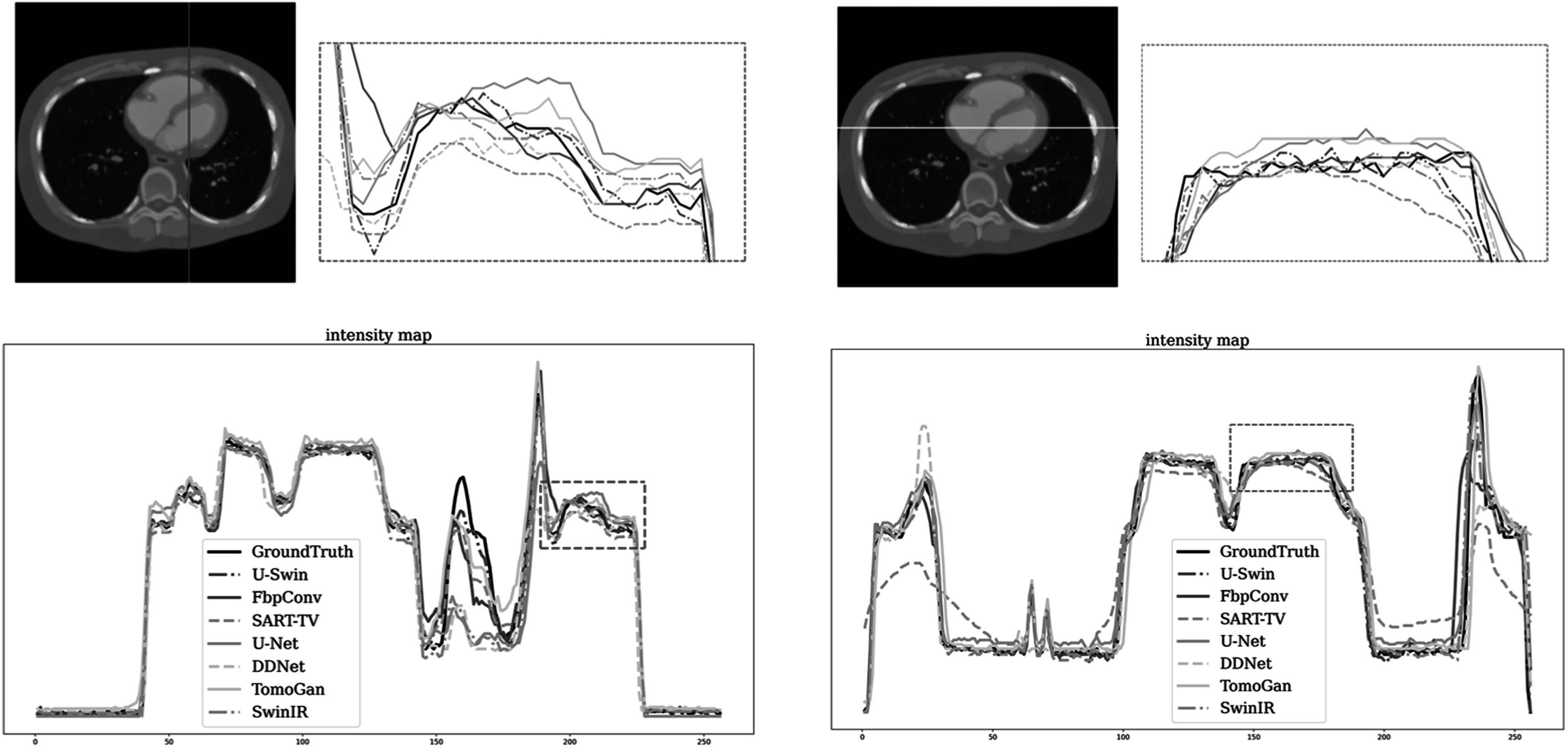
Representative intensity profiles of various methods on a slice of patient 153 (phase 40, slice 21). Particularly, the top left figure indicates the slice location, and the top right figure showcases a magnified view of the region of interest. Notably, the top right figure features a zoomed-in view on the region of interest.

**Figure 9.**
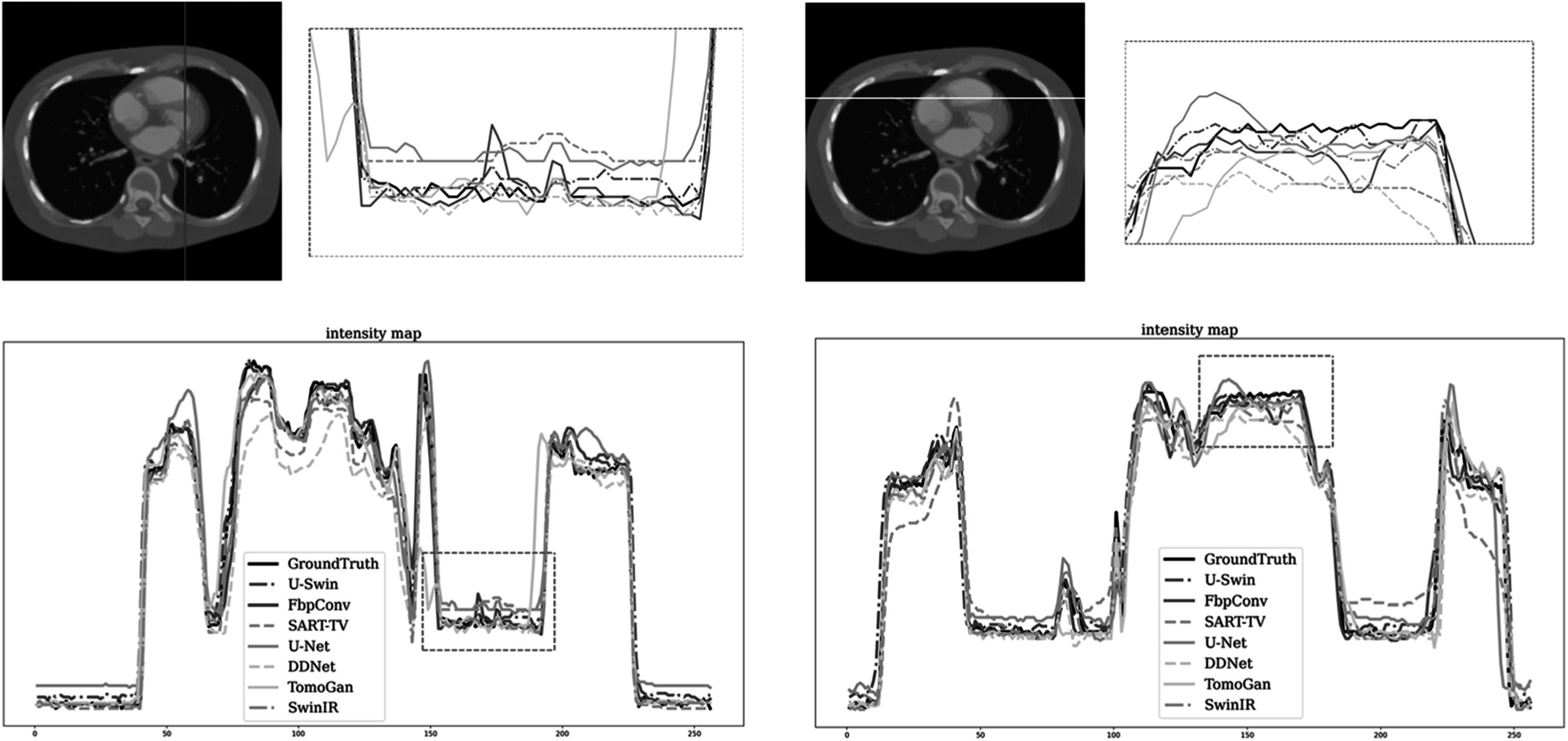
Same as figure 8 but for patient 153 (phase 40, slice 10).

To further investigate the comparison among different methods, we plot representative profiles along line segmentations to conduct a detailed analysis of edge preservation and noise levels. We choose two representative lines within the image, one vertical and one horizontal. As shown in figure 8, the proposed method exhibits lower volatility compared to the ground truth, particularly in regions with higher gradients, indicating better edge information recovery. In regions with a small gradient, our method demonstrates minimal differences and reduced gradients, indicating the presence of less noise. To further validate our findings, we also present results from the second slice, as illustrated in figure 9.

**Figure 10.**
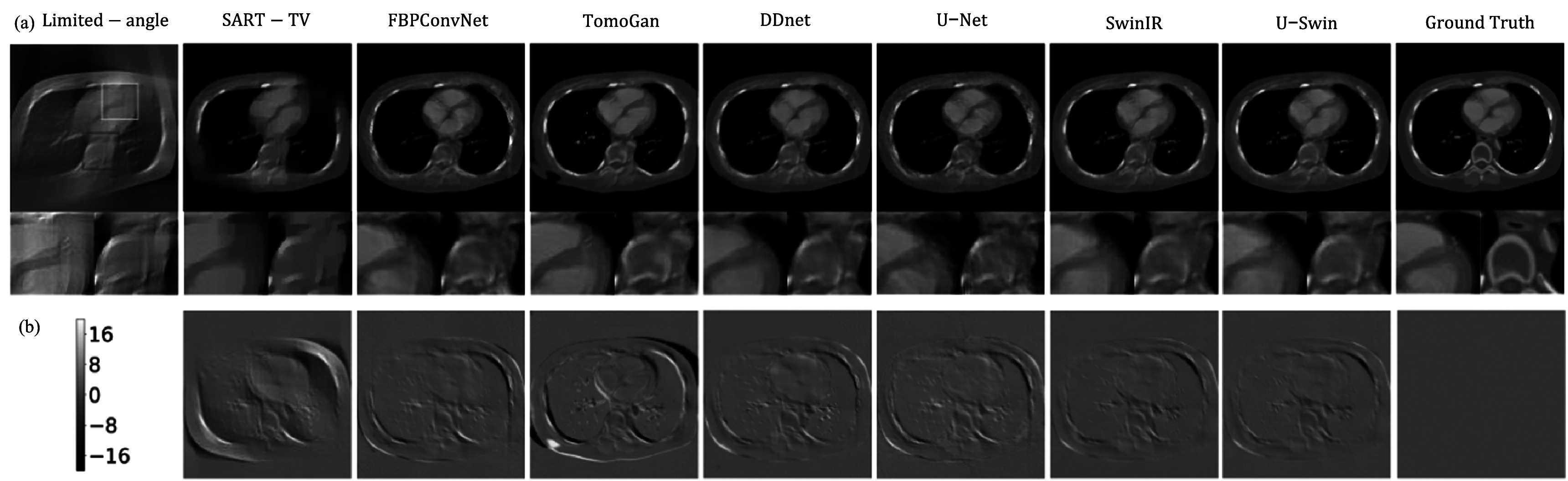
Reconstructed results of a representative image slice from patient 153 (phase 52, slice 17). (a) Visual comparison of cone-beam CT reconstructions generated from 90 degree viewing angles using different methods. The last column shows the ground truth (GT) images. Each reconstructed image is accompanied by two magnified ROIs (regions of interest). The display window is [−800, 1000] HU. (b) The difference maps between reconstructed and ground truth images of each method in terms of HU.

When the angular range is reduced to a smaller range, the limited-angle reconstructed images have more severe streak artifacts, and it is more challenging to reconstruct high quality images. However, our proposed method still outperforms other methods, and it can restore clearer boundaries with less noise. Please see representative results from 90 degree projections in figure 10 and quantitative evaluation results in table 1.

**Table 1.** Quantitative comparison (average RMSE/SSIM) on the testing dataset.

		Limited angle	SART-TV	FBPConvNet	U-Net	TomoGan	DDNet	SwinIR	U-Swin
120°	SSIM	0.6772	0.8365	0.9381	0.9409	0.9357	0.9424	0.9487	**0.9617**
	RMSE	0.0089	0.0028	0.0016	0.0016	0.0018	0.0014	0.0015	**0.0009**
90°	SSIM	0.5278	0.8489	0.9073	0.8736	0.8815	0.9215	0.9398	**0.9409**
	RMSE	0.0103	0.0031	0.0019	0.0020	0.0032	0.0018	0.0016	**0.0015**

We compare the reconstruction results quantitatively on the testing dataset using SSIM and RMSE. The average values of SSIM and RMSE for each method are summarized in table 1. Higher SSIM values and lower RMSE values indicate superior image quality in the reconstruction results. Among all the methods, the traditional iterative reconstruction method SART-TV exhibits the lowest SSIM value. Notably, the SwinIR model outperforms FBPConvNet, U-Net, DDNet and TomoGan. For the angular range of 120°, our proposed method increases SSIM value by 0.013 compared to the SwinIR and reduces RMSE by 35.7% compared to DDNet. For the angular range of 90°, our proposed method has higher SSIM value and smaller RMSE values than all other approaches. The quantitative measures of reconstruction confirm that our proposed method outperforms other competing methods.

### 4.5. Ablation study

To thoroughly evaluate the proposed hybrid U-Net transformer framework and validate the performance under different settings, a variety of ablation studies are performed, including: (1) the skip-connection and U-Net block, (2) sequence length and patch size, and (3) model scaling.

As the aforementioned, incorporating U-Net-like skip connections can restore additional low-level spatial information, thereby potentially improving image reconstruction performance. Those connections are alongside the skip connection within the UST module, which fuse transformer blocks and U-Net blocks. The image reconstruction performance is summarized in table 2. We can see that adding skip-connection generally leads to a better reconstruction performance. Consequently, we adopt this configuration for our U-Net transformer. We then test the image reconstruction performance using the Swin-transformer without U-Net block. Based on our limited-angle experiments, we observe a slower convergence rate due to the presence of missing data in many areas, making it a challenge for computation. In the experiments where transformer blocks are excluded, the model is transitioned to a convolution-based architecture. The absence of an attention mechanism results in reduced global information, leading to a lower overall performance. These results reinforce our initial intuition to integrate U-Net-like skip-connections into the Transformer design to enable learning precise low-level details.

**Table 2.** Quantitative evaluation of ablation study.

	SSIM	RMSE
U-Swin w/o skip connection	0.9334	0.0017
U-Swin w/o U-Net block	0.9428	0.0011
U-Swin w/o transformer block	0.9422	0.0014
U-Swin	0.9631	0.0009

We also explore the impact of patch size on the proposed network. Our observations indicate that a smaller patch size leads to superior image reconstruction performance compared to a larger patch size. The transformer’s sequence length is determined by the number of patches, inversely proportional to the square of the patch size. Consequently, a smaller patch size, or an increase in the effective sequence length, demonstrates robust improvements. This is because the Transformer can encode more complex dependencies between each element for longer input sequences. In line with the ViT setting, we adopt an 8×8 patch size as the default in our experiments.

We conducted an ablation study to assess the impact of different model sizes in U-Swin. The network size is controlled by the following parameters: hidden channel size, number of UST modules, number of transformer blocks in each UST module, and the number of heads in each transformer block. As shown in table 3, ‘U-Swin1’ incorporates a single UST block along with one transformer block, while ‘U-Swin-Light’ features two consecutive UST blocks, each accompanied by a transformer block. We further explore two configurations: the ‘U-Swin-Small’ and ‘U-Swin-Large’ models. In the ‘Small’ model, the hidden channel size, number of UST modules, number of transformer blocks, and number of heads are set to 30, 3, 3, and 6, respectively. Correspondingly, for the ‘Large’ model, these parameters are set to 60, 6, 6, and 6. From table 3, we observe that a larger model contributes to improved performance, with U-Swin-Large exhibiting notably higher SSIM values and lower RMSE values, showcasing its compatibility with enhanced image quality. The model can be constructed to be even larger, but this may result in an overfitting issue. As a result, considering both performance and GPU card capacity, we chose to implement the larger model in our application.

**Table 3.** The comparison between model size and performance.

	Block number	SSIM	RMSE
U-Swin1	(1)	0.9344	0.0017
U-Swin-Light	(1,1)	0.9248	0.0018
U-Swin-Small	(3,3)	0.9433	0.0016
U-Swin-Large	(6,6)	0.9631	0.0009

### 4.6. Clinical limited-angle CT results

**Figure 11.**
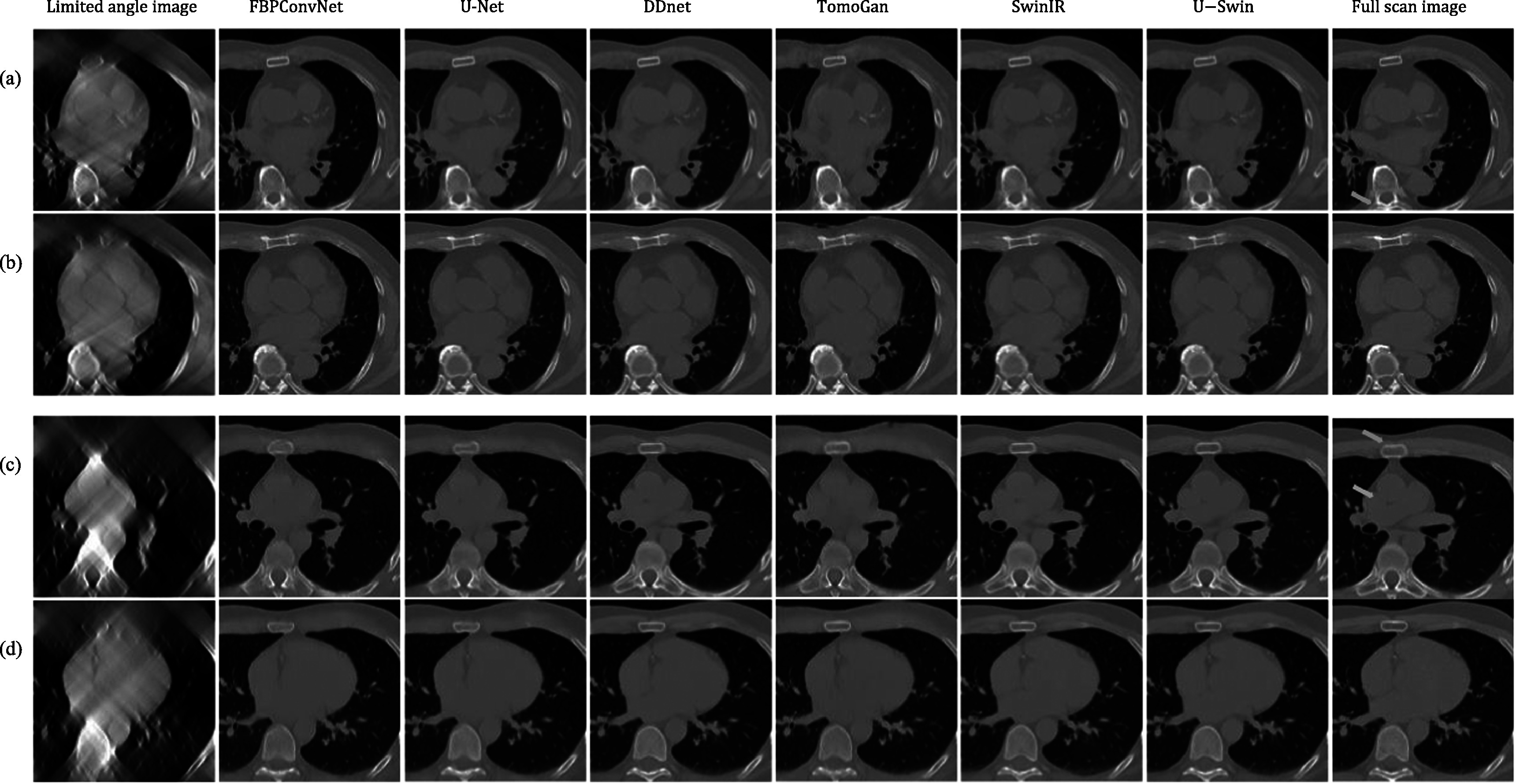
Representative image slices reconstructed from limited-angle projections in the COCA dataset. (a) and (b) are slices 18 and 23 of patient 99 from 120° angular range projections. Rows (c) and (d) are slices 9 and 22 of patient 123 from 90° angular range projections. Each row shows the comparison between different network reconstruction results. The display window is [−900, 1000] HU.

In this part, we compare the reconstruction results of FDK, convolution-based model FBPConvNet, transformer-based model SwinIR and our proposed U-Swin model, and other competing methods, on the real cardiac CT image dataset COCA. The limited-angle dataset contains 120° and 90° projections. The limited-angle reconstruction images, full view reconstruction images, and the corresponding reconstructed images using different networks are shown in figure 11. Here, two different slices of cardiac CT images are compared for both 120° and 90° projections. All the methods successfully suppress severe streak artifacts and reconstruct the image with high quality. In regions where most information is lost, our proposed method excels in reconstructing images that closely resemble the ground truth as indicated by the arrow in figure 11. The quantitative evaluation results are summarized in table 4. Comparing the original limited-angle reconstruction image for the angular range of 120°, which only achieves a SSIM value of 0.6021 and a relatively large RMSE of 0.1334, each method significantly enhances the image quality. In contrast to FBPConvNet and SwinIR, our proposed method demonstrates an improvement in SSIM by 0.006 and 0.0027, respectively, while reducing RMSE values by 7.6%. Comparing to DDNet, our method performs better for and 90° but lightly worse for 120°. More comprehensive analysis and experiments will be done in the follow-up studies.

**Table 4.** Quantitative comparison (average RMSE/SSIM) on the COCA dataset.

		Limited angle	FBPConvNet	U-Net	TomoGan	DDNet	SwinIR	U-Swin
120°	SSIM	0.6021	0.9385	0.9442	0.9289	0.9474	0.9418	0.9446
	RMSE	0.1334	0.0258	0.0220	0.0302	0.0221	0.0239	0.0229
90°	SSIM	0.5471	0.8876	0.9114	0.8693	0.9096	0.9137	0.9145
	RMSE	0.1380	0.0297	0.0295	0.0485	0.3049	0.0325	0.0293

In table 5, it shows that transformer-based models require more parameters compared to convolution-based models. This is because the self-attention mechanism in transformers utilizes three matrices (for queries, keys, and values) to capture long-range dependencies and contextual information, leading to an increased parameter number and a higher computational cost.

**Table 5.** Comparison of number of parameters between different models.

Model	FBPConvNet	U-Net	TomoGan	DDNet	SwinIR	U-Swin
params	67.86 M	54.70 M	26.87 M	25.6 M	125.17 M	129.63 M

## 5. Discussions and conclusion

Limited-angle CT is valuable to enhance temporal resolution and reduce motion artifacts but optimization of image quality in this scenario remains highly challenging. To address this issue, deep learning techniques have been developed to improve cardiac CT image quality. While the U-Net excels in segmentation tasks, the transformer partitions an image into patches, capturing more global information through a self-attention mechanism. Our proposed limited-angle reconstruction approach combines U-Net and Swin-transformer blocks to establish a novel network architecture that restores missing information effectively. Specifically, we integrate the U-Net and Swin-Transformer to form a UST block, and then incorporate two UST blocks into the overall architecture. In the previous work (Liu *et al* 2021), stacking more transformer blocks was reported to improve the network performance, but this will increase the number of parameters dramatically and computational cost accordingly. Our architecture has optimized the number of blocks empirically and demonstrated to produce more accurate results more efficiently.

Concerning the quality of the reconstructed images, the vessels and structural information are faithfully restored. For example, edges along various directions are clear, without severe image noise and artifacts. Our network clearly outperforms other methods in terms of restoring bone and organ structures. However, our research concentrates on two specific angular sizes and starting angles, which may limit the robustness. We believe that there is a potential to further suppress image noise and enhance overall image quality by introducing the diffusion model-based prior, which will be reported in our follow-up studies.

In conclusion, we have proposed a U-Swin network to combine the advantages of Swin- transformer and U-Net to solve the limited-angle topographic reconstruction problem. We have provided the detailed network structures and demonstrated its effectiveness. By comparing the reconstructed image with SART-TV, FBPConvNet, DDnet, U-Net, and TomoGan trained on the same simulated XCAT dataset and clinical COCA dataset 2021, we have shown that our methodology can effectively eliminate the severe streak artifacts and enhance the reconstructed image quality with higher SSIM and smaller RMSE values.

## Data Availability

The data cannot be made publicly available upon publication because they are not available in a format that is sufficiently accessible or reusable by other researchers. The data that support the findings of this study are available upon reasonable request from the authors.
